# Servant Leadership and Followers Prosocial Rule-Breaking: The Mediating Role of Public Service Motivation

**DOI:** 10.3389/fpsyg.2022.848531

**Published:** 2022-07-05

**Authors:** Naqib Ullah Khan, Muhammad Zada, Asad Ullah, Afraseyab Khattak, Heesup Han, Antonio Ariza-Montes, Luis Araya-Castilo

**Affiliations:** ^1^School of Public Administration, Central South University, Changsha, China; ^2^Business School, Henan University, Kaifeng, China; ^3^Department of Management Sciences, Alhamd Islamic University, Islamabad, Pakistan; ^4^Institute of Business and Management Studies, The University of Agriculture, Peshawar, Pakistan; ^5^College of Hospitality and Tourism Management, Sejong University, Seoul, South Korea; ^6^Social Matters Research Group, Universidad Loyola Andalucía, Córdoba, Spain; ^7^Facultad de Economía y Negocios, Universidad Andrés Bello, Santiago de Chile, Chile

**Keywords:** servant leadership, prosocial rule-breaking, public service motivation, public managers, corona relief tiger force

## Abstract

This research explores the effect of servant leadership on prosocial rule-breaking (PSRB) and the mediating mechanism of public service motivation (PSM) between the association of servant leadership and PSRB. The said phenomenon is examined in the civil service context of Pakistan during the continuing crises of the COVID-19 pandemic, a situation where the traditional civil service policy and rule system has become highly complicated for passionate employees’ service performance and efficiency, and where servant leadership has received greater attention for inspiring the attitudinal and behavioral outcomes of frontline workers during the pandemic. Data were collected from 546 frontline workers of the corona relief tiger force. The findings of the study revealed that servant leadership has a significant effect on PSRB and PSM, and that PSM significantly promotes PSRB. The results also revealed that servant leadership has a significant impact on PSRB *via* engendering PSM.

## Introduction

Organizations formulate and execute policies and rules to streamline employees’ behaviors toward efficient service provision and achieving goals (Borry and Henderson, 2020). Uncertainty and dynamic changes in the external environment continuously challenge the efficiency of the prevailing policies and rules systems of organizations. Particularly, the catastrophic effects of the pandemic continue to affect various service organizations in different ways (Finsterwalder and Kuppelwieser, 2020). Some organizations have completely shut down service provision while the work burden of essential service organizations including civil service agencies has increased globally (Gofen and Lotta, 2021). Civil service agencies continue to execute the traditional service policies and rules systems and are simultaneously responsible to implement pandemic-related measures for preventing its adverse socio-economic impacts. A stream of literature has evidence that formal policies and rules are not always productive instead sometimes hinder performance efficiency, particularly when there is uncertainty and dynamic changes in the external environment (Vardaman et al., 2014; Ghosh and Shum, 2019; Zada et al., 2021). Lipsky (2010) demonstrates that even well-articulated and most comprehensive rules are not always sufficient to overcome the intricacies of the workplace. Shum et al. (2019) have argued that the existing rules of some organizations restrict employees’ flexibility for an efficient service provision. Consistently, Dahling et al. (2012) have argued that employees break formal organizational policies and rules to facilitate the stakeholders of the organization with the intention that such rule-breaking will improve efficiency or the overall image of the organization. Such rule violating behavior is referred to as prosocial rule-breaking (PSRB) in organizational management. PSRB is defined as: “an intentional violation of formal policies and rules to promote organizational welfare or one of its stakeholders” (Morrison, 2006). PSRB has numerous positive individual and organizational outcomes including promoting cooperation among employees and with other stakeholders of the organization (Dahling et al., 2012), and improving organizational performance, efficiency, and development (Morrison, 2006; Ghosh and Shum, 2019). PSRB is important for leaders and managers as it indicates that the existing policy and rule system has shortcomings in resolving the needs and problems of the workplace (Fleming, 2020). The literature has evidence that individuals’ characteristics for promoting PSRB are well debated, however, limited and inconclusive studies are available on the situational antecedents of PSRB.

Although, the intentions behind PSRB are positive, rule-breaking has associated risks of criticism and punishment. Therefore, a prosocial rule-breaker passes through an uncertain situation as expects admiration for contributing beyond responsibilities and simultaneously expects criticism for violating rules (Morrison, 2006). Social information processing theory (SIPT) (Salancik and Pfeffer, 1978) posits that an individual actively evaluates an outer element before engaging in behaviors that have an associated risk of punishment. As leadership holds the highest powers to criticize or punish rule-breaking. According to SIPT, an employee actively evaluates the leadership approach before engaging in risky behaviors (Salancik and Pfeffer, 1978). Positive leadership behavior is considered a key situational factor for influencing followers’ attitudes and behaviors (Langhof and Güldenberg, 2020; Hameduddin and Engbers, 2021) including employees’ risky voice (Lapointe and Vandenberghe, 2018) and PSRB behavior as well (Zhu et al., 2018; Wang and Shi, 2020). Compared to other positive leadership behaviors, servant leadership is considered a unique follower and community-focused leadership behavior (Greenleaf, 1977). Servant leadership has several followers centered attributes including keeping close one to one interpersonal relationships with followers (Greenleaf, 2002), taking care of followers’ personal and professional interests (Washington et al., 2006), allowing and empowering followers to make independent decisions for problems-solving (Van Dierendonck, 2011), and satisfying followers’ emotional, ethical, spiritual, and relational needs (Lee et al., 2020). Servant leadership has received special attention for encouraging and influencing followers’ behavioral outcomes during the present pandemic situation (Fernandez and Shaw, 2020; Hu et al., 2020). According to SIPT, servant leadership may promote followers’ engagement in PSRB, because of the follower’s experiences and perceptions that servant leadership with servant characteristics will admire or at least not criticize PSRB behavior. Using SIPT, this study uncovers the potential effect of servant leadership on PSRB behavior.

The literature has evidence that leadership not only directly influences the followers’ behaviors but also through altering and activating the followers’ attitudes and motivations (Walumbwa et al., 2010). Public service motivation (PSM) is an important concept and construct of public management, which has received increased attention from public management scholars and practitioners with special relevance to public administration (Piatak and Holt, 2021). Consistently, state-owned enterprises (Papenfuß and Keppeler, 2020) public sector volunteers as well as non-profit public service organizations (Costello et al., 2020) where the goal of service delivery is to contribute to society. PSM is a dynamic condition influenced by various organizational forces (Perry and Wise, 1990), and managerial leadership is one of the most important organizational interventions which is consistently suggested to practitioners for improving PSM (Hameduddin and Engbers, 2021) and as an alternative to the traditional means (e.g., pay for performance) for fostering PSM in public and non-profit workers (Papenfuß and Keppeler, 2020). Social psychological theories, such as the role modeling effects of social learning theory (SLT) (Bandura and Walters, 1977) are used for supporting the association between managerial leadership and followers PSM (Fareed and Su, 2021). Some limited studies have noted that servant leadership with servant characteristics fosters employee PSM (Shim and Park, 2019; Tran and Truong, 2021), and only one study has noted that employees with high PSM are more likely to engage in PSRB (Weißmüller et al., 2020). The dimensions of PSM such as public interest and civic duty; compassion; and self-sacrifice (Perry, 1996) may encourage rule-breaking for prosocial reasons. “Moreover, the existing literature on PSM demonstrates that the ‘antecedents and outcomes of PSM are not very consistent” (Ritz et al., 2016; Chung et al., 2021). Therefore, scholars emphasize additional studies from different samples in different contexts to enhance the generalizability of the concept of PSM (Bawole et al., 2019). Particularly, there is a lack of any empirical evidence on how servant leadership influences PSRB *via* engendering PSM.

The present study was different from the earlier studies as it uncovers the relationship between servant leadership with PSRB using SIPT. Similarly, the study explored the mediating role of PSM in the said relationship while using SIPT and SLT. Moreover, this study re-examined the previously explored association of servant leadership with PSM and PSM with PSRB in a special sample of corona relief tiger force (CRTF) in the continuing catastrophic situation of COVID-19.

## Theory and Hypotheses

### Servant Leadership and Prosocial Rule-Breaking

The concept of servant leadership is the focus of research for a few decades (Greenleaf, 1977), but this leadership style has received special attention for research as one of the most important leadership approaches for inspiring and promoting followers outcomes of frontline workers to provide a better response during the ongoing crises of the pandemic (Fernandez and Shaw, 2020; Hu et al., 2020; Ngoma et al., 2020), where the frontline workers are serving multiple responsibilities in an uncertain environment that is affecting their emotional and psychological wellbeing (Tecco, 2020). Servant leadership is defined as the “servant-leader is servant first, it begins with the natural feeling that one wants to serve, to serve first” (Greenleaf, 1977). Compared to other leadership styles, servant leader has unique followers and others-centered attributes. For example, servant leadership keeps close interpersonal relationships with followers and listens to and fulfills their personal and professional needs (Greenleaf et al., 2002; Washington et al., 2006); a servant leader promotes the well-being of followers and others (Laub, 1999). Moreover, servant leadership emphasizes the importance of followers by appreciating, respecting, listening, and empowering them (Stone et al., 2004); it is considered a self-scarifying behavior in the interest of followers and other stakeholders of the organization (Barbuto and Wheeler, 2006). Servant leadership provides feelings of trust, acceptance, and empowerment (Van Dierendonck, 2011). Compared to other leadership approaches servant leadership is a high potential source of satisfying followers based on emotional, ethical, spiritual, and relational grounds with an ultimate focus to accomplish organizational goals (Lee et al., 2020). This leadership style promotes creativity and improves problem-solving skills through the provision of an opportunity for high interpersonal interaction and communication (Van Dierendonck et al., 2014), and empowers followers to make independent decisions and experiment with new procedures for problem-solving (Eva et al., 2018). The literature has evidence that servant leadership produces servant followership through learning and role modeling (Wu et al., 2021), and that servant leadership encourages risky behaviors such as voice behavior at the workplace (Lapointe and Vandenberghe, 2018; Arain et al., 2019). Servant leadership is found as one of the most important leadership behavior for promoting followers’ outcomes including prosocial behaviors during the continuing crises of the COVID-19 pandemic (Hu et al., 2020; Wang and Yin, 2020).

Researchers have classified rule-breaking behaviors based on motives behind such behaviors (self vs. others). Self-centered rule-breaking behaviors such as workplace deviance (Galperin, 2012) and counterproductive behaviors (Whelpley and McDaniel, 2016) are considered negative and highly undesirable as here someone breaks policies and rules for personal gains at the expense of the organizational interests. Unlike self-centered rule-breaking, PSRB is referred to as an intentional violation of policies, rules, and norms to forward the interests of the organization and its stakeholders (Morrison, 2006). Along the same lines, Dahling et al. (2012) argue that employees break formal rules with the intention to promote the service efficiency of an organization. Employees break formal rules for facilitating colleagues, clients, citizens, and other stakeholders of an organization to meet their expectations from the organization. Fleming (2020) demonstrates that frontline civil servants have high work burdens and are required to make quick decisions to provide civil services. The pandemic has particularly increased their service demands as they have to serve multiple responsibilities including traditional civil service provision and additional services to averse to the adverse socio-economic impacts of the pandemic. Therefore, frontline civil servants are highly likely to break policies and rules for multiple prosocial reasons, including facilitating each other to ensure civil service provision on schedule, facilitating clients and citizens during work breaks for improving service efficiency and organizational image building, etc. Studies have also evidence that the frontline civil workers are even more aware of the concerns and needs of the clients and citizens than the policymakers of the civil service agencies (Lipsky, 2010; Borry and Henderson, 2020). Thus, there are multiple avenues for frontline workers to break formal policies and rules for various prosocial reasons. Earlier studies have noted that job autonomy, fellow workers’ rule-breaking (Morrison, 2006), high morality (Vardaman et al., 2014), and high-risk propensities (Kahari et al., 2017) are some of the factors that encourage PSRB. Managerial leadership is considered one of the key factors for promoting PSRB (Zhu et al., 2018; Wang and Shi, 2020). The existing literature emphasizes additional studies to explore other aspects and approaches of managerial leadership for promoting PSRB. The pandemic has particularly alerted leadership scholars and practitioners to find appropriate leadership behaviors for stimulating followers’ prosocial behaviors.

Social psychological theories are widely used for explaining the relationships between leadership behaviors and followers’ outcomes. SIPT (Salancik and Pfeffer, 1978) posits that individuals’ attitudinal and behavioral actions are influenced by individual processing and evaluation of information received from the external environment. Leaders hold the most powerful positions in organizations, therefore followers actively evaluate the behavior of their leaders, and followers then shape their attitudinal and behavioral actions according to the potential response from their leaders (Lau and Liden, 2008). Dahling et al. (2012) demonstrate that the members of an organization increase and decrease PSRB based on the perceptions of admiration or criticism, respectively, from their leaders. If followers perceive that the leader will admire or at least not punish PSRB, then the followers will engage in PSRB, while on the other hand, if the risk of punishment is high, then the followers will disengage from PSRB. A servant leader has several followers and others-centered attributes such as altruistic calling, listening, empathy, healing, awareness, persuasion, empowerment, foresight, stewardship, growth, and community building (Barbuto and Wheeler, 2006). Therefore, the followers of a servant leader may perceive that the importance of PSRB will be understood by the leader in the wider organizational interest. Based on SIPT, we assume that servant leadership with servant characteristics promotes PSRB. The following hypothesis is presented for testing:

H1: Servant leadership relates to prosocial rule-breaking.

### Servant Leadership and Public Service Motivation

Public service motivation was initially defined as “an individual predisposition to respond to motives grounded primarily or uniquely in public institutions and organizations” (Perry, 1996). However, PSM exists in public sector volunteers as well as non-profit organizations (Park and Kim, 2016; Costello et al., 2020) where the goal of service delivery is to contribute to the good of others or the society, it might matter socially oriented businesses as-well (McCarthy et al., 2019). The construct of PSM has four distinct components such as commitment to the public interest and civic duty; compassion; self-sacrificing behavior (altruism); and attraction toward policy-making (Perry, 1996). The dimensions of PSM such as commitment to the public interest, compassion, and self-sacrificing attitudes are directly related to actions taken in the interest of the public and others. Earlier studies have noted managerial leadership as one of the key factors for inspiring and promoting PSM (Schwarz et al., 2020; Fareed and Su, 2021; Iqbal and Ahmad, 2021; Iqbal and Piwowar-Sulej, 2021). Compared to other leadership styles servant leadership has more unique followers and other-centered attributes, including altruistic calling, listening, empathy, emotional healing, awareness, persuasion, empowerment, foresight, stewardship, growth, and community building (Barbuto and Wheeler, 2006); emphasis on moral and ethical values (Graham, 1991); self-sacrifice, compassion, and altruism (Eva et al., 2019); followers satisfaction based on emotional, ethical, spiritual, and relational grounds (Lee et al., 2020). The characteristics of servant leadership such as; altruism, compassion, moral and ethical considerations, self-sacrificing behavior, and caring for others’ interests lead directly toward the components of PSM such as altruism and compassion toward the public; commitment toward public interest; and self-sacrificing for others. Wu et al. (2021) argued that servant leadership behavior is actively learned by the followers of a servant leader. SLT by Bandura and Walters (1977) posits that behaviors are acquired and learned both through vicarious reinforcements (e.g., rewards and punishments) and observation, imitation, and role modeling as well, even in the absence of direct reinforcements (Bandura and Walters, 1977). Hunter et al. (2013) argued that leadership behaviors are even more actively learned and imitated by followers because of the power and status held by leaders in organizations. Therefore, we assume that the followers and others focused attributes of servant leadership may encourage others focused PSM of followers through learning and role modeling. Schwarz et al. (2016) demonstrate that servant leadership promotes PSM in public sector employees. Some studies have shown that the antecedents of PSM are not very consistent (Ritz et al., 2016; Hameduddin and Engbers, 2021), therefore, there is a need for further studies in different contexts to enhance the generalizability of the construct of PSM (Bawole et al., 2019). Using SLT, this study examines the association of servant leadership and PSM in the context of Pakistan. The following hypothesis is developed for analysis:

H2: Servant leadership relates to public service motivation.

### Public Service Motivation and Prosocial Rule-Breaking

Employees break rules for stakeholders of an organization including colleagues, clients, citizens, and others to serve and resolve their problems with an ultimate aim to benefit the organization (Dahling et al., 2012). Researchers demonstrate that organizational policies and rules are not always productive to solve complexities of the workplace (Lipsky, 2010; Kumar Bandyopadhyay et al., 2020) and that the existing rules of some organizations restrict employees’ flexibility for an efficient service provision (Shum et al., 2019; Sun et al., 2020). The frontline civil workers are even more aware of the problems than the policy and rule makers of public agencies themselves (Fleming and Bodkin, 2018; Borry and Henderson, 2020). Thus, employees are always highly likely to engage in PSRB behavior for others which ultimately leads to wider organizational interests. Perry (1996) argues that employees with high PSM have specific public service tendencies such as improving the existing policies in the public interest; compassionate feelings to facilitate others; commitment to serving the public; and self-sacrificing propensity to promote the public interest. Thus, as employees with public service motives are working for the common good of the society, and PSRB is a risky behavior incurred to facilitate others in the wider organizational interest, therefore it is highly likely that other-focused PSM may promote other focused PSRB, specifically in an environment where employees perceive that the leader of the organization has followers and others-centered attributes as-well such as servant leadership. One study has particularly noted that employees with high PSM actively engage in PSRB behavior (Weißmüller et al., 2020). Using the propositions of SIPT, we assume that high PSM promotes PSRB. The following hypothesis is developed for analysis:

H3: Public service motivation relates to prosocial rule-breaking.

### Mediating Effect of Public Service Motivation

The existing literature has widely discussed PSM as an important mediating mechanism between managerial leadership and sub-ordinate employees’ performance and behavioral outcomes in various contexts (Ritz et al., 2016; Bawole et al., 2019; Hameduddin and Engbers, 2021). Some studies have particularly noted that servant leadership improves employees’ job performance (Schwarz et al., 2016) and innovative work behavior (Askaripoor et al., 2020) through the mediating mechanism of PSM. However, the existing literature has lacked any empirical research evidence on how servant leadership influences PSRB *via* PSM. Using the propositions of SLT, we assume that the followers and others-centered attributes of servant leadership engender others focused PSM, and high PSM then ultimately promotes PSRB. The relationships among these variables can also be explained in the light of SIPT as the evaluation of the others (e.g., followers and others) focused attributes of servant leadership inspire others oriented PSM, which ultimately improves others focused PSRB. The following hypothesis is developed for analysis:

H4: Public service motivation mediates the relationship of servant leadership with prosocial rule-breaking.

## Materials and Methods

### Sample and Procedures

At the end of March-2020 soon after the emergence of the COVID-19 pandemic in Pakistan, the prime minister of Pakistan has mobilized the youth of the country (e.g., including students, social workers, teachers, doctors, etc.) to form CRTF with an initial purpose to facilitate and coordinate with the government and civil administration to contain the spread of the pandemic and to mitigate the undesirable socio-economic impact of the pandemic. Currently, the members of CRTF are actively performing various duties and relief activities including creating awareness regarding social distancing and implementation of the standard operating procedures (SOPs) formulated to contain the spread of the pandemic, implementation of Ehsaas (care) and Panagah (shelter) welfare programs, regulating prices of food items and distribution of foods in the needy and poor people, monitoring working of utility stores, checking hoarding and mitigating climate change through the implementation of billion tree plantation drive, etc. The members of CRTF are working under district administrations and under indirect supervision and control of deputy and assistant commissioners (civil service officials) across Pakistan. To collect data from the members of CRTF for this study, a plan was set in September-2020 to invite and attract 600 members of the force using various sources including the offices of district administration officials (e.g., Islamabad, Pindi, Peshawar, Mardan, Charsada, Bannu, Kohat), various social media platforms/groups of CRTF (e.g., “Corona Relief Tiger Force”; “Tiger Force”; “Prime Ministers Tigers Force ICT Islamabad Office,” etc.) and using the support of friends working with the force themselves. After 2 months in November-2020, we managed to receive the willingness of 600 members of CRTF to participate in the surveys of the study. All these members were included in What’s App groups using their phone contacts provided by district management offices and members of the force themselves in person, through friends, and on social media. The invitation, attraction, and selection of all these members for the study are on voluntary bases, on the condition of confidentiality, and using convenient sampling procedures.

Surveys for this study were completed online at two different time intervals, to minimize the risk of common method variance associated with data collection through self-reported measures sometimes (Podsakoff et al., 2003). The first phase of the study was completed in the middle of November, and 560 out of the 600 members of CRTF have provided valid responses to the measures of the independent variable (servant leadership) and demographic variables of the study. After 1 month in the middle of January-2021, 546/560 members of CRTF provided valid responses measuring the dependent variable (PSRB) and mediating variable (PSM). Each participant received a fixed number/id through which the 2-stage data were matched, and all those 546 members who have provided valid responses were from the 560 members who have validly completed the first phase of the study. Based on the initially planned target of participants’ selection for this study, the final valid response rate of the participants was 91%. The demographics of the 546 respondents finalized for analyses were as: 335 (61%) were male, 234 (42%) were married, and 478 (87.54%) were having undergraduate and above level education. The average age of respondents was 28.13 years (SD = 6.21), and the work experience of the respondents was uniform, as all the respondents joined CRTF in April 2020 when the force was first established by the government of Pakistan.

### Measures

Variables of the study were measured through standardized multi-item scales adapted from previous literature. All responses were measured using a 5-point Likert scale, where “1” stands for strongly disagree, and “5” for strongly agree.

### Servant Leadership

Servant leadership was measured using the 7-item scale developed by Liden et al. (2015). Some items of the scale are “My leader can tell if something work-related is going wrong,” “My leader gives me the freedom to handle difficult situations in the way that I feel is best,” and “My leader puts my best interests ahead of his/her own.” This 7-item scale is one of the most widely used measures of servant leadership (Eva et al., 2019), which is also used in the Asian (Miao et al., 2014), and Pakistani public sectors as-well (Abid et al., 2015; Arain et al., 2019). Cronbach’s alpha (α) reliability of the scale was 0.9, and values of the second-order confirmatory factor analysis (CFA) of the scale were [χ^2^(14) = 41.15, χ^2^/*df* = 2.74, *p* < 0.001, CFI = 0.96, TLI = 0.95, RMSEA = 0.06].

### Public Service Motivation

Public service motivation was measured using the 5-item scale developed by Wright et al. (2012). Examples of the items are “Meaningful public service is very important to me,” “I am often reminded by daily events about how dependent we are on one another,” and “I am prepared to make enormous sacrifices for the good of society.” This scale is adopted from the seminal work of Perry (1996) and is widely used in earlier studies (Ritz et al., 2016; Choi and Chung, 2018), including in studies from Pakistan (Quratulain and Khan, 2015). The Cronbach’s alpha and second-order CFA values of the scale were [α = 0.89, χ^2^(5) = 13.67, χ^2^/*df* = 2.95, *p* < 0.05, CFI = 0.95, TLI = 0.93, RMSEA = 0.07].

### Prosocial Rule-Breaking

Prosocial rule-breaking was measured using the 13-item scale developed by Dahling et al. (2012). Some examples of the items are “I break organizational rules/policies to do my job more efficiently,” “I disobey organization regulations that result in inefficiency for the organization,” and “I assist other employees with their work by breaking organizational rules.” This scale is recently tested in Pakistan by Asadullah et al. (2019) and in the subcontinent of India and Pakistan by John and Shafi (2020). The Cronbach’s alpha and the second-order CFA values of the scale were [α = 0.92, χ^2^(52) = 139.18, χ^2^/*df* = 2.18, *p* < 0.001, CFI = 0.97, TLI = 0.94, RMSEA = 0.05].

### Control Variables

We included gender, age, and marital status of the study participants as control variables according to former studies (Morrison, 2006; Dahling et al., 2012). Previous studies have taken organizational tenure and type as well as controls. We excluded them for further analyses as the sample of this study is members of CRTF, who have uniform tenure and work only for civil service agencies.

### Data Analysis

Data of the study were analyzed using SPSS 22, Amos 23, and PROCESS macro. Reliability analyses, descriptive statistics, and correlational analyses were performed using SPSS. Degrees of discrimination amongst the variables of the hypothesized model was analyzed using CFAs in Amos. The assumptions of regression were confirmed with testing for receiving the evidence of normality and linearity, and with the absence of multi-collinearity and heteroscedasticity, before hypotheses testing. Hypotheses of the study were analyzed using simple linear regression and mediation model 4 using SPSS-PROCESS macro 3.3 proposed and developed by Hayes (2017).

## Results

### Confirmatory Factor Analysis

Confirmatory factor analysis was used to confirm whether the three-factor model (servant leadership, PSRB, and PSM) fits the data. The results showed that the three-factor model is well fitted to the data [χ^2^ = 415.76, *df* = 203, χ^2^/*df* = 2.048, *p* < 0.001, CFI = 0.91, TLI = 0.93, RMSEA = 0.04]. Compared to the hypothesized three-factor model of the study, the other constrained models (two-factor, and one-factor models) were having a poor fit. The lack of common method bias was confirmed through the application of Harman’s single factor test, as the value of the highest emerging un-rotated first factor was 34.4%, which indicates that the data has no issues of common method bias, as the extracted value was under the critical points of 50 and 40% highlighted by Podsakoff and Organ (1986) and Fuller et al. (2016), respectively. Data of all the three latent constructs (servant leadership, PSRB, and PSM) were analyzed for composite reliability (CR) and convergent validity. As indicated in Table 1, the values of CR for all the three constructs were above 0.8, which infers that the constructs of the study have excellent internal consistencies (Fornell and Larcker, 1981; Netemeyer et al., 2003). Convergent validities of the constructs were confirmed through average variance extracted (AVE). As shown in Table 1, the values of AVEs for all the three constructs were above 0.5, which infers that the constructs have no issues of convergent validity (Sarstedt and Cheah, 2019). As shown in Table 1, all the correlation statistics are in the expected directions. For example, servant leadership is positively and significantly correlated with PSRB (*r* = 0.52, *p* < 0.01), and PSM (*r* = 0.38, *p* < 0.01). PSM and PSRB are also positively and significantly correlated (*r* = 0.21, *p* < 0.01). The preliminary analyses conclude that the scales, modeling, and data of the study are processed accurately. These initial analyses encourage us to process and evaluate the data for hypothesis testing.

**TABLE 1:** Descriptive statistics, composite reliability, and convergent validity.

Variables		CR	AVE	Mean ± SD	1	2	3	4	5
1	PSRB	0.91	0.58	4.17 ± 0.80	–				
2	SL	0.90	0.75	3.81 ± 0.37	0.52**	–			
3	PSM	0.88	0.62	3.49 ± 0.65	0.21**	0.38**	–		
4	Gender	–	–	0.64 ± 0.51	0.10	−0.13	0.24*	–	
5	Marital status	–	–	0.68 ± 0.45	0.02	−0.05	0.04		–
6	Age	–	–	28.13 ± 098	0.04	0.06	0.08	0.04	0.05

SL, servant leadership; PSRB, pro-social rule-breaking; PSM, public service motivation; CR, composite reliability; AVE, average variance extracted; SD, standard deviation. *p < 0.05, **p < 0.01.

### Hypotheses Testing/Analyses

Hypotheses of the study were analyzed through SPSS-PROCESS-macro 3.3 developed by Hayes (2017). Simple linear regression and mediation model-4 were used for extracting the results of the hypothesized relationships. As indicated in Table 2, results revealed that servant leadership was significantly related to PSRB (β = 0.74, SE = 0.03, *p* < 0.001), and PSM as well (β = 0.51, SE = 0.04, *p* < 0.001). PSM was also significantly related to PSRB (β = 0.32, SE = 0.02, *p* < 0.001). The mediating effect of servant leadership on PSRB *via* PSM was extracted using mediation model-4 at 5000 bootstraps and 95% CI. Results revealed that the indirect effect of servant leadership on PSRB *via* PSM was (β = 0.16, BootSE = 0.02, *p* < 0.001 LLCI = 0.12, ULCI = 0.22). As indicated 0 does not fall between the lower and upper bounds of the bootstraps intervals, which infers that the indirect effect of servant leadership on PSRB *via* PSM is significant (Hayes, 2018). This implies that servant leadership influences PSRB to an extent of 0.16 (12%) *via* the mediating mechanism of PSM. Moreover, all the control variables were insignificantly related to PSRB and PSM, however, only gender was significantly related to PSM (0.19^**^, *p* < 0.01), which suggests that PSM is high in males than in their female counterparts. The regression analyses conclude support for all the hypotheses H1, H2, H3, and H4 of the study.

**TABLE 2:** Regression results.

			Product of β				Boot/95% CI	
		
Path			β	SE	*t*	*p*	Lower	Upper
SL	→	PSRB	0.74	0.03	23.33	<0.001	0.68	0.80
SL	→	PSM	0.51	0.04	13.65	<0.001	0.44	0.59
PSM	→	PSRB	0.32	0.03	9.48	<0.001	0.25	0.38
SL → PSM	→	PSRB	0.16	0.02	7.79	<0.001	0.12	0.22

*N = 546, where N represents number of respondents.*

*SL, servant leadership; PSM, public service motivation; PSRB, prosocial rule-breaking; β, coefficient; SE, standard error; t, t-value.*

## Discussion

Organizations formulate policies and rules to streamline employee behaviors to achieve organizational goals. Essential civil service organizations have currently imposed multiple complex policies and rules for ensuring traditional civil services and for preventing the adverse socio-economic impacts of the pandemic (Gofen and Lotta, 2021). Researchers demonstrate that even well-established policies and rules have always shortcomings and that the frontline workers are even more aware of the needs and wicked problems of the societies than the policymakers themselves (Fleming, 2020; John and Shafi, 2020). Therefore, there are multiple avenues for rule-breaking for the frontline workers to break rules for prosocial reasons. In such circumstances, the high passionate employees sometimes break the established policies and rules with intentions and motivations to facilitate colleagues, clients, citizens, and other stakeholders of organizations to improve performance efficiency or to build organizational image (Dahling et al., 2012; Fleming, 2020). Rule-breaking with positive intentions, such as to serve the stakeholders of an organization with an ultimate focus to promote service efficiency or organizational image, is referred to as PSRB (Morrison, 2006). PSRB is exposed to both admirations for working extra and criticism for rule-breaking. The leadership of an organization holds the most powerful position in an organization (Iqbal et al., 2020, 2021c). Therefore an employee who engages in PSRB must think about the potential response of admiration or criticism from the leader before engaging in such behavior (Iqbal et al., 2021a; Wang and Shi, 2021). As servant leadership has unique followers, communities, and other focused attributes such as satisfaction of employees on all the ethical, emotional, spiritual, and relational grounds to promote organizational interests (Lee et al., 2020), servant leaders listen to the opinions of followers through keeping close interpersonal relationships (Greenleaf, 1977), and it is a self-sacrificing leadership behavior to promote the interests of followers and other stakeholders (Barbuto and Wheeler, 2006); servant leadership provides feelings of trust, acceptance, and empowerment (Van Dierendonck, 2011). Moreover, servant leadership has been recognized as an encouraging leadership behavior in the present pandemic for inspiring the behavioral outcomes including prosocial behaviors of employees (Hu et al., 2020). Using SIPT, this study has noted that servant leadership significantly encourages PSRB behavior. It is possibly because of the followers’ perceived experiences that servant leadership with servant characteristics may admire or at least not criticize unselfish rule-breaking for others, which promotes the larger interests of the civil service organizations. Some earlier studies have also noted that other positive managerial leadership approaches such as ethical leadership (Zhu et al., 2018) and inclusive leadership (Wang and Shi, 2021) promote PSRB. Our study adds to the previous literature by finding that servant leadership is an important external situational factor for promoting PSRB.

Employees with high PSM have specific predispositions such as tendencies to reform public policies, commitment to the public interest, compassionate behaviors, and self-sacrificing and altruistic behaviors to promote the public interest. PSM is a public sector-specific motivation where someone with high PSM is highly likely to contribute toward common social good through the facilitation and cooperation of others including the general public. Using SLT, this study has noted that servant leadership significantly influences and promotes others’ focused PSM. This finding is in line with earlier studies that found that servant leadership is a predictor of PSM (Askaripoor et al., 2020; Tran and Truong, 2021). Moreover, so for only one study has evidenced that PSM is a predictor of PSRB (Weißmüller et al., 2020). Using the propositions of SLT and SIPT, our study has also noted that PSM significantly promotes PSRB. Reconfirming this result in the present circumstances meets our expectations correctly that other-centered PSM is a push toward employees’ engagement in PSRB in an environment of rule complexity. As systematic reviews show that the antecedents and outcomes of PSM are not very consistent (Ritz et al., 2016; Hameduddin and Engbers, 2021), therefore (Bawole et al., 2019) have encouraged to conduct of more studies on PSM from different samples in different contexts for enhancing the generalizability of the construct of PSM. The findings of our study from a unique sample of CRTF and a different context of Pakistan encourage us that servant leadership can be believed as a significant predictor of PSM, and that PSM is an encouraging factor of PSRB.

Moreover, researchers demonstrate that managerial leadership not only directly influences followers’ behavioral and organizational outcomes but also through altering the attitudes and motivations of followers. Employee PSM is found as an important mediating mechanism between managerial leadership behaviors and followers’ behavioral and organizational outcomes in public and non-profit settings (Papenfuß and Keppeler, 2020; Wang and Yin, 2020; Hameduddin and Engbers, 2021). So far, there is a lack of any empirical research evidence on how servant leadership influences PSRB *via* the mediating mechanism of PSM. Using SLT, our study has noted that servant leadership significantly influences PSRB, *via* the mediating mechanism of PSM. That is servant leadership engenders PSM, which ultimately improves PSRB. Some studies have also noted that servant leadership encourages job performance (Schwarz et al., 2016) and innovative work behaviors (Askaripoor et al., 2020), *via* the mediating mechanism of PSM. This evidence encourages us that servant leadership can be believed to be an important managerial leadership for promoting PSM, which ultimately enhances PSRB.

### Theoretical and Practical Implications of the Study

This study has two important theoretical contributions. Using SIPT, this study has noted for the first time, that servant leadership significantly influences PSRB. With this finding, this study extends the servant leadership approach to the emerging literature on prosocial behaviors, particularly the literature seeking external situational factors for PSRB (Fleming, 2020; Wang et al., 2021). Using SLT, the second most important contribution of this study is the finding that PSM works as an internal mediating mechanism between the association of servant leadership and PSRB. That is servant leadership engenders PSM, which ultimately promotes PSRB. This empirical evidence extended the servant leadership literature to the literature on PSRB through an intervening mechanism of PSM. Moreover, earlier studies have empirical research evidence between servant leadership and PSM (Tran and Truong, 2021), and between PSM and PSRB (Weißmüller et al., 2020). However, some scholars believe that the antecedents and outcomes of PSM are not always consistent (Ritz et al., 2016; Hameduddin and Engbers, 2021). Therefore, it is encouraged to investigate the antecedents and outcomes of PSM in different contexts from different samples. Our study shows that servant leadership significantly predicts PSM and that PSM significantly promotes PSRB. These findings are received from a unique sample of CRTF in the context of Pakistan. This means that the relationships among these variables are stable. Therefore, we are encouraged to believe that servant leadership is an important external situational factor for promoting PSM, and that high PSM encourages PSRB.

This study has several practical implications. As stated employees, PSRB becomes highly essential for frontline civil workers to ensure efficient civil service provision (Fleming, 2020; John and Shafi, 2020), particularly in situations when civil servants are operating under a complex policy and rule environment as the continuing crises of the pandemic. Highly passionate frontline workers know the needs and problems of societies more than the policy and rule makers themselves, and therefore frontline workers as CRTF may break the formal policies and rules to ensure efficient service provision. PSRB is not only highly important for employees of an organization to facilitate one another and other stakeholders of an organization (Dahling et al., 2012; Weißmüller et al., 2020), but organizational performance efficiency, development, and image building (Morrison, 2006), but also for leaders and managers for understanding deficiencies in the policy and rule system of an organization (Fleming, 2020). We encourage inducting employees into the civil service systems with attributes such as empathy, self-sacrificing behaviors, cognitive consciousness, and proactive personalities as such employees are highly likely to act independently to engage in PSRB, when required (Mayer et al., 2007; Dahling et al., 2012). More, PSM is considered an alternative to paying for performance in public and non-profit organizational settings (Papenfuß and Keppeler, 2020; Iqbal et al., 2021b). We encourage inducting employees into the civil service systems with high public service motives such as tendencies toward policy-making, commitment to the public interest, compassion, and self-sacrificing behaviors (Perry and Wise, 1990). Employees with high PSM are highly likely to work passionately in crisis situations such as the continuing pandemic situation, and PSM is an encouraging factor in PSRB behavior. Therefore, we encourage keeping PSM a key criterion of selection for new hires in public agencies.

Moreover, this study has found servant leadership as an important external situational factor for encouraging both PSM and PSRB. Therefore, we encourage policymakers, leaders, and managers to promote servant leadership specifically in civil service agencies. Managers with attributes such as altruism, morality, listening, emotional healing, awareness, persuasiveness, foresightedness, stewardship, and well-being of others (Barbuto and Wheeler, 2006; Lee et al., 2020) can work as servant leaders. Candidates with such potential servant leadership attributes can be recruited and selected during the hiring process. These characteristics of servant leadership can also be taught in training and development programs for civil servants. Civil servants can further be monitored to execute servant leadership effectively. Finally, we encourage to use of servant leadership more often than usual during the continuing situations of the pandemic, as this way we may promote other-oriented PSM and PSRB in the civil service providers of public agencies.

### Limitations and Future Research Directions

This study is conducted using a commonly known quantitative self-report method. The main strengths of this method are that it allows a large number of participants to quickly and easily report and describe their own experiences and feelings rather than inferring from observing participants. Using the self-report technique, we measured the subjective experiences and thoughts of respondents regarding servant leadership, PSM, and PSRB. The limitation of the study can be that we measured PSM and PSRB using self-report measures answered by the sampled respondents themselves. The main limitation of self-reporting PSM and PSRB can be that participants may want to present themselves in a socially acceptable manner, social desirability bias can be a problem with self-report measures as participants may want to portray themselves in a good light. However, social desirability bias was minimized by ensuring confidentiality and anonymity of responses. The second limitation of the study is that all the responses were collected from the same sources, which sometimes causes common method bias (Podsakoff et al., 2003). However, the risk of common method bias was reduced by collecting data at two different points’ intervals, and the Harmons single factor test has shown that there was a lack of common method bias. In the future, we encourage collecting data from different sources such as managers to respond to the measure of PSRB and followers to measure servant leadership. Future studies may use three staged data such as measuring servant leadership, PSM, and PSRB at three different points’ intervals. The mixed-method approach can be used to develop a more in-depth understanding of the established relationships. Morrison (2006) has argued that PSRB toward performance efficiency, colleagues, and community has different intensity. Therefore, servant leadership can be tested with each dimension of PSRB to develop a more in-depth understanding of the research phenomenon. The association between servant leadership and PSRB can be tested in other contexts and different samples to further improve the generalizability of the findings. Authentic leadership behavior can be tested with PSRB as it’s also a positive leadership behavior and some of its attributes are the same as servant leadership (Hoch et al., 2018), therefore authentic leadership can also be important leadership behavior for encouraging PSRB.

## Conclusion

This research aims to examine the impact of servant leadership on PSRB and to examine the mediating mechanism of PSM between the association of servant leadership and PSRB. These associations are examined in the civil service context of Pakistan during the continuing crises of the pandemic, a situation where the traditional service policy and rule system have become highly complex for passionate employees to contribute effectively and efficiently, and where servant leadership behavior has received increasing attention for research for promoting followers’ behavioral outcomes. Results of the study revealed that servant leadership significantly promotes followers’ PSM and PSRB and that PSM significantly influences PSRB. Results also revealed that servant leadership promotes PSRB *via* engendering PSM.

## Data Availability Statement

The original contributions presented in this study are included in the article/supplementary material, further inquiries can be directed to the corresponding authors.

## Author Contributions

NK, MZ, and AU contributed to the conception, design of the database, performed the statistical analysis, and wrote the first draft of the manuscript. AK, HH, AA-M, and LA-C wrote sections of the manuscript. All authors contributed to manuscript revision, read, and approved the submitted version.

## Conflict of Interest

The authors declare that the research was conducted in the absence of any commercial or financial relationships that could be construed as a potential conflict of interest.

## Publisher’s Note

All claims expressed in this article are solely those of the authors and do not necessarily represent those of their affiliated organizations, or those of the publisher, the editors and the reviewers. Any product that may be evaluated in this article, or claim that may be made by its manufacturer, is not guaranteed or endorsed by the publisher.
